# Monitoring exercise-induced muscle damage indicators and myoelectric activity during two weeks of knee extensor exercise training in young and old men

**DOI:** 10.1371/journal.pone.0224866

**Published:** 2019-11-07

**Authors:** Zoltán Heckel, Tamás Atlasz, Éva Tékus, Tamás Kőszegi, József Laczkó, Márk Váczi

**Affiliations:** 1 Doctoral School of Health Sciences, Faculty of Health Sciences, University of Pécs, Pécs, Hungary; 2 Department of Sportbiology, Institute of Sport Sciences and Physical Education, University of Pécs, Pécs, Hungary; 3 János Szentágothai Research Centre, University of Pécs, Pécs, Hungary; 4 Department of Laboratory Medicine, Medical School, University of Pécs, Pécs, Hungary; 5 Department of Informatics and Biorobotics, Institute of Mathematics and Informatics, University of Pécs, Pécs, Hungary; 6 Department of Theory and Practice of Sports, Institute of Sport Sciences and Physical Education, University of Pécs, Pécs, Hungary; University of L'Aquila, ITALY

## Abstract

This study considered the effects of repeated bouts of short-term resistive exercise in old (age: 64.5±5.5 years; n = 10) and young men (age: 25.1±4.9 years; n = 10) who performed six knee extension exercise bouts over two weeks using various markers of exercise-induced muscle damage and electromyographic activity. We found that time-course changes in quadriceps isometric torque, creatine kinase activity, and muscle soreness in the two groups were similar. However, recovery in the acute torque deficit was mediated by more favourable electromyographic activity changes in the young group than in the older adults group. Muscle elastic energy storage and re-use assessed with dynamometry was selectively improved in the young group by the end of the protocol. Serum myoglobin concentration increased selectively in old group, and remained elevated with further bouts, suggesting higher sarcolemma vulnerability and less effective metabolic adaptation in the older adults, which, however, did not affect muscle contractility.

## Introduction

Aging induces-muscle wasting (sarcopenia) and strength loss are well-known processes. By the age of 70 years, the skeletal muscle cross-sectional area and muscle strength are reduced by 25–30% and 30–40%, respectively [1]. The loss of muscle strength continues to decrease by 1–2% per year [2]. Scientists and gerontologists design intervention programs to target general weakness, and it has been recommended that high-intensity resistive exercise be used to delay sarcopenia but may be limited due to muscle injury and repair [3].

While high force (especially eccentric-biased) exercise has strength- and growth-promoting effects in skeletal muscle [4,5], the high tension applied during such contractions may induce myofibrillar disruption, delayed onset muscle soreness, elevation of muscle proteins in serum, and temporary strength loss in both young and old humans [6,7,8,9,10]. These exercise-induced muscle damage (EIMD) markers usually peak between 24–48 hours after exercise and recover between days 5 and 7 in antigravitational muscles [11].

The responsiveness to a single bout of eccentric exercise has been extensively investigated in young and old humans with varying outcomes that may be the result of different methodologies of EIMD quantification. For example, evidence exists that old humans are more susceptible to EIMD compared to young humans, and recovery is delayed after a single bout of eccentric exercise when inflammatory markers are measured [12,13]. In contrast, studies using the conventional indirect EIMD markers (serum enzymes and proteins, strength, soreness) have demonstrated greater damage in young versus old subjects [14,15].

The repeated bout effect has also been studied, and it is generally accepted that, regardless of age, EIMD markers show less severe changes after the second exercise bout, compared with the first [16,17]. The phenomenon is often explained by neural adaptation [18,19], allowing the muscle to better distribute the workload among fibres; however, more recent studies have demonstrated that reduced inflammation [20] or remodelling of the extracellular matrix [21] may contribute to protection against a second injury. The effect of age on the repeated bout effect was studied by Lavender & Nosaka [15], using indirect EIMD markers, and it was demonstrated that when the two eccentric exercise bouts were performed with a four-week hiatus, the protective effect conferred by the first bout was less pronounced in the old group. These results were confirmed by Gorianovas et al. [14], who applied two stretch-shortening cycle exercise bouts separated by two weeks. In contrast to these studies, a similar repeated bout effect was found in young and old women when the first eccentric exercise bout was repeated seven days after the first [7]. The inconsistency in the outcomes of these studies may originate from several factors, such as different experimental designs, different criterion measures, and different ages and genders of the subjects studied.

Two important problems emerge from the studies of how long the protective effect lasts in older adults. First, despite the fact that protection may play a role in early neuroadaptation, no myoelectric activity measurements were made [9,22]. Second, the recovery periods between bouts used in the experiments were unrealistic; therefore, results are less informative when a systematic exercise intervention program is to be designed to target muscle weakness. Numerous studies demonstrate that three sessions per week is optimal for ageing individuals for the development of muscle strength and size [4,23,24]. Though evidence exists that muscle strength can increase in as short as two weeks (six sessions) of resistance training in the older adults [25,26], experiments do not provide clear evidence about the time-course of EIMD and early recovery/adaptation in young versus ageing muscle when the bouts are systematically repeated.

Another important issue in the EIMD studies is that a loss of acute performance was observed in muscle mechanical properties such as dynamic and isometric force, rate of force development and stretch–shortening cycle (SSC) dynamics [27,28,29]. However the magnitude of the deficit and the recovery time, after a single exercise bout, varied. Furthermore the time course of loss of performance, recovery and adaptation were not similar during an 11-day-long exercise program using seven bouts [22]. Ageing-induced impairment of the aforementioned mechanical properties raises problems in the older adults by increasing risks of falls and mobility disability, and impairment of these mechanical properties is thought to be related to sarcopenia [30,31,32]. Thus, we propose that, in the early phase of exercise, the magnitude of adaptations in different mechanical properties are age-dependent because old individuals demonstrated a greater loss in rapid force producing capacity after four days of lower limb disuse and an incomplete recovery in all strength properties during the seven-day active period compared to young controls [33].

In the present study, a two-week-long quadriceps exercise intervention consisting six bouts was designed to examine the short-term adaptability of young and old human muscles. Specifically, we tested the hypotheses that: (i) the time-course of changes in EIMD markers (serum protein levels, isometric torque, muscle soreness) and myoelectric activity are different in young versus old subjects, and (ii) the two-week changes in rate of torque development (RTD) and SSC function are different in young versus old subjects.

## Materials and methods

### Subjects

Ten healthy, physically active young men (age: 25.1±4.9 years, height: 176±6.9 cm, weight: 72.4±17.6 kg) and older adults men (age: 64.5±5.5 years, height: 176.2±8.8 cm, weight: 80.3±10 kg) participated in the study. The older adults group was a group of acquaintances of the university, whereas the young subjects were students. Subjects underwent a medical screening and completed training and a health status questionnaire before the beginning of the study. Major exclusion criteria were as follows: current knee injuries, previous hip surgery, and existing muscle pain, endocrine disorders such as diabetes, cardiovascular diseases, and pelvic inflammatory disease. The subjects were advised to avoid any vigorous physical activity or unaccustomed exercises, to maintain their normal dietary and sleep habits, and not to take any anti-inflammatory drugs (e.g. non-steroidal anti-inflammatory agent) or nutritional supplements (e.g. vitamins, protein/amino acids) during the experimental period. One week before the first day of the investigation, the subjects reported to the laboratory for a familiarisation session during which they were acquainted with the testing equipment. Subjects gave written informed consent according to the Declaration of Helsinki after receiving both a verbal and a written explanation of the experimental protocol and its potential risks. The present study was approved by the University of Pecs Ethical Committee. (file number: 4817).

### Design and procedures

Subjects performed six eccentric-concentric exercise bouts over two weeks (Table 1). There were five test sessions: before bout 1, and at 24 h, 48 h, one week and two weeks after bout 1 (Table 1). In these sessions, we assessed maximal voluntary isometric (MVC) torque and the myoelectric (EMG) activity of the quadriceps femoris muscle, and we also measured the levels of different serum markers. All exercises and tests were performed in the morning (between 9:00 and 12:00). Blood samples were always taken before exercise or test sessions. In test sessions 1 and 5, we also determined the RTD and SSC function.

**Table 1:** Exercise bouts, test sessions, and tests/measurements performed during the experiment.

	Day 1 (Test 1)	Day 2 (Test 2)	Day 3 (Test 3)	Day 4	Day 5	Day 6	Day 7	
Serum markers	*	*	*					
Isometric MVC	*	*	*					
SSC function	*							
Muscle soreness	*	*	*					
Exercise bout	*		*		*			
	**Day 8** (Test 4)	**Day 9**	**Day 10**	**Day 11**	**Day 12**	**Day 13**	**Day 14**	**Day 15** (Test 5)
Serum markers	*							*
Isometric MVC	*							*
SSC function								*
Muscle soreness	*							*
Exercise bout	*		*		*			

Data obtained from test session 1 were considered as baseline data.

MVC = maximal voluntary contraction

SSC = stretch-shortening cycle

### Mechanical muscle properties

Multicont II dynamometer (Mediagnost, Budapest and Mechatronic Ltd., Szeged, Hungary) was used for testing the mechanical properties of the quadriceps muscle. Subjects were seated on the padded seat of the dynamometer and performed three maximal voluntary isometric contractions (MVCs) at 70° of knee flexion (0° = full extension). Subjects were instructed to generate the highest possible torque as quickly as possible. Peak torque and RTD were determined offline from the torque–time curves. RTD was quantified as RTD (Nm/ms) = dM (Nm) / dt (ms), where M is the torque and t is the time in milliseconds. RTD was determined for the first 30-, 50-, 100-, and 200-ms intervals from the onset of the contraction. The greatest values obtained from these measurement intervals were averaged for every subject and were used for statistical analysis.

MVC torque was also measured at a 30° knee angle to determine the trigger threshold for initiating the SSC test contraction. To determine SSC function, subjects performed a quadriceps SSC contraction during which the dynamometer rapidly applied a preset amount of energy to stretch the quadriceps [22]. The eccentric phase of the contraction started at 30° of knee flexion and the subject had to exert force against the lever arm as fast and forcefully as possible. When the subject reached 60% of isometric MVC torque measured previously at 30° of knee flexion, the lever arm of the dynamometer started to rotate in the direction of knee flexion. Subjects were instructed to resist the rotating lever arm maximally, stop it within the shortest range of motion (eccentric phase), and then extend the knee without a time delay and as quickly as possible to 30°. The initial velocity of the lever arm was 300°·s^-1^ and the preset amount of stretch-load (expressed in Joules) was half of the baseline isometric MVC torque. The applied stretch-load represents the amount of work the lever arm of the dynamometer performed on the shank to flex the knee joint. As the eccentric knee flexion progresses, the energy stored in the servomotor diminishes to zero (the lever arm stops) and some of the energy is stored in the quadriceps muscle. The instructions given to the subjects ensured that the transfer of energy that stretched the quadriceps muscle occurred in a short time and over a small range of motion so that the concentric contraction (i.e., knee extension) would start without delay. During the concentric phase, the dynamometer motor was automatically turned off and provided resistance through friction and the inertia of the lever arm and lower leg (S1 Video). Torque and knee angle as a function of time were recorded for each contraction, and similar to Kyröläinen et al. [34], we calculated the negative (Wssc^-^) and positive mechanical work (Wssc^+^) during the SSC by integrating the torque–position curve between the boundaries of the range of motion:

$$W(J)=\int_{\theta_{1}}^{\theta_{n}}M_{\left(\theta \right)}\cdot d\theta$$

where M = torque, dθ = angular displacement, and θ1 and θn represent the first and the last knee angle data points, respectively. Total time to complete the eccentric (Tssc^-^) phase, the concentric phase (Tssc^+^), and the entire SSC contraction (Tssc), were also determined.

To examine the ability to store and re-use elastic energy, a pure concentric contraction was performed after the SSC test. For each subject, this contraction started exactly at the knee angle where the dynamometer lever was stopped during the SSC test (transition phase) and ended in the 30° position. Subjects fully relaxed their quadriceps and then performed maximal effort knee extension during which, similar to the concentric phase of the SSC described above, the dynamometer motor was turned off, and provided resistance through friction. For this contraction, mechanical work (Wcon) was calculated as in the equation above. To investigate the ability to store and re-use elastic energy, positive SSC work and the pure concentric work ratio (Wssc^+^/Wcon) were determined.

### Serum markers

Ten-milliliter blood samples were collected from an antecubital vein by a standard vein puncture technique using disposable needles, and were placed in Vacutainer plain tubes (Becton-Dickinson). After clotting, the blood sample was centrifuged at 1500 *g* for 10 min to obtain serum. Serum were stored at -80°C until being analysed for creatine kinase (CK) activity and myoglobin (Mb) concentration. CK activities were determined using a standard routine laboratory test (kinetic optimized UV method, COBAS INTEGRA^®^ 400 plus, Roche Diagnostics GmbH, Mannheim, Germany). Using this methodology, the reference range of CK was 0–200 IU·l^-1^. For Mb determination, an automated chemiluminescence immunoassay was applied (Immulite 1000, Siemens Healthcare Diagnostics GmbH, Marburg, Germany). The upper reference limit at 97.5 percentile for Mb was 70 μg L^-1^; however, based on the manufacturer’s suggestions, the median value was 25 μg L^-1^.

### Muscle soreness

Subjects reported the soreness level sensed during the MVC test contractions. Subjects were asked to mark a point on a visual analogue scale of 50 mm in length, where 0 mm signified “no pain” and 50 mm signified “extremely painful”. The length of the line from 0 to the marked point provided a numeric measure of soreness [18].

### EMG activity

Surface electromyography (EMG) signals were recorded (1000 Hz sampling rate) from the vastus lateralis (VL) and vastus medialis (VM) muscles (Noraxon, Scottsdale, USA). The skin was carefully prepared by shaving, rubbing, and cleaning with alcohol. Dual Ag/AgCl electrodes (Noraxon, Scottsdale, USA) with a 20-mm inter-electrode distance were placed over the muscle belly, in accordance with SENIAM recommendations (www.seniam.org). The raw EMG signals were rectified, filtered and smoothed using the root mean square (RMS) method with a 200 ms smoothing window. The peak EMG activity of VL and VM were averaged and used for data analysis.

### Dynamometric exercise

The exercise bouts consisted of maximal effort knee extensions performed on the dynamometer as described previously. Subjects trained using the right limb only. Each bout started with a warm-up of five minutes of cycling on a cycle ergometer, followed by stretching the knee extensor muscles. After warming up, subjects performed 4 sets of 15 repetitions eccentric-concentric contractions at 60°∙s^-1^ constant angular velocity over 60° of range of motion, between 20° and 80° of knee joint position. Subjects were encouraged to maximally resist the dynamometer’s rotating lever arm during the eccentric phase and then to extend the knee forcefully during the concentric phase. A one-second rest was provided between repetitions and a two-minute rest was provided between sets. Peak torques achieved during the exercise contractions were averaged for every subject in every bout. Verbal encouragement was given to subjects during the exercise.

### Statistical analysis

Descriptive statistics (mean values and standard deviations) were computed for the measured and calculated variables. All variables were checked for normality. Between-group differences in the baseline values were determined using independent t-tests (all mechanical variables, EMG activity) and Mann-Whitney U tests (CK, Mb). Exercise-induced changes in all mechanical variables and EMG activity were analysed using a two-way (group by time) mixed model analysis of variance (ANOVA). In case of significant interaction or time main effects, the Bonferroni correction was used for post-hoc analysis to perform pairwise comparisons. We determined the training effects across time in dependent variables such as Mb and CK using a nonparametric Friedman ANOVA. To test differences in these variables, the Wilcoxon matched pairs test was used for post hoc analysis. Because muscle soreness was measured on the ordinal scale, differences were determined using the nonparametric Mann-Whitney U test. The significance level was set at p<0.05.

## Results

Barring the Wssc^+^/Wcon ratio, the baseline values for the mechanical variables and EMG activity were significantly greater in the young group (p<0.05). There was no between-group difference in baseline CK activity and Mb level.

A significant between-group difference was found in the exercise torques achieved during the six bouts (young = 224 ± 37 Nm; old = 177 ± 37 Nm; p = 0.001).

**Fig 1:**
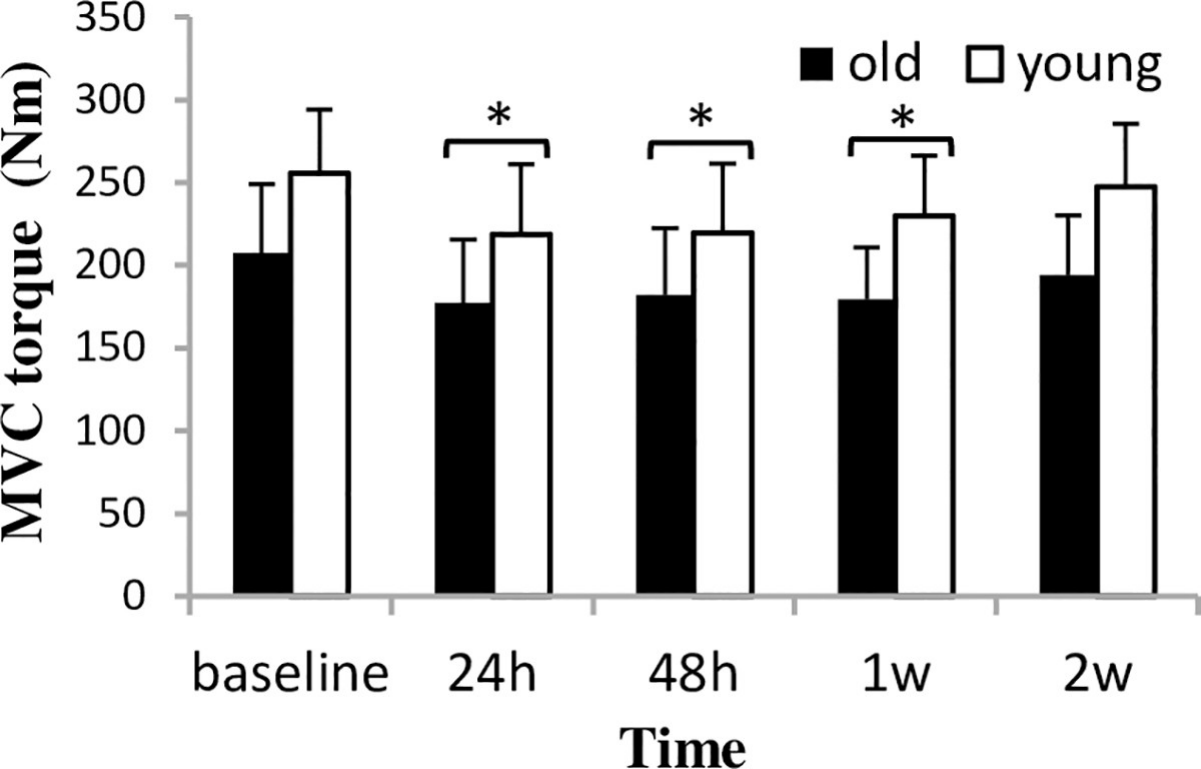
MVC torque (mean±SD) changes during a two-week eccentric-concentric knee extensor training in old and young men. MVC = maximal voluntary contraction * Significantly different from baseline (p<0.05) after significant time-main effect.

A significant time main effect was found for MVC torque (F_4.15_ = 20.03 p = 0.000), without group by time interaction. MVC torque decreased uniformly in the two groups 24 h after the first bout and returned to baseline after two weeks (Fig 1).

**Fig 2:**
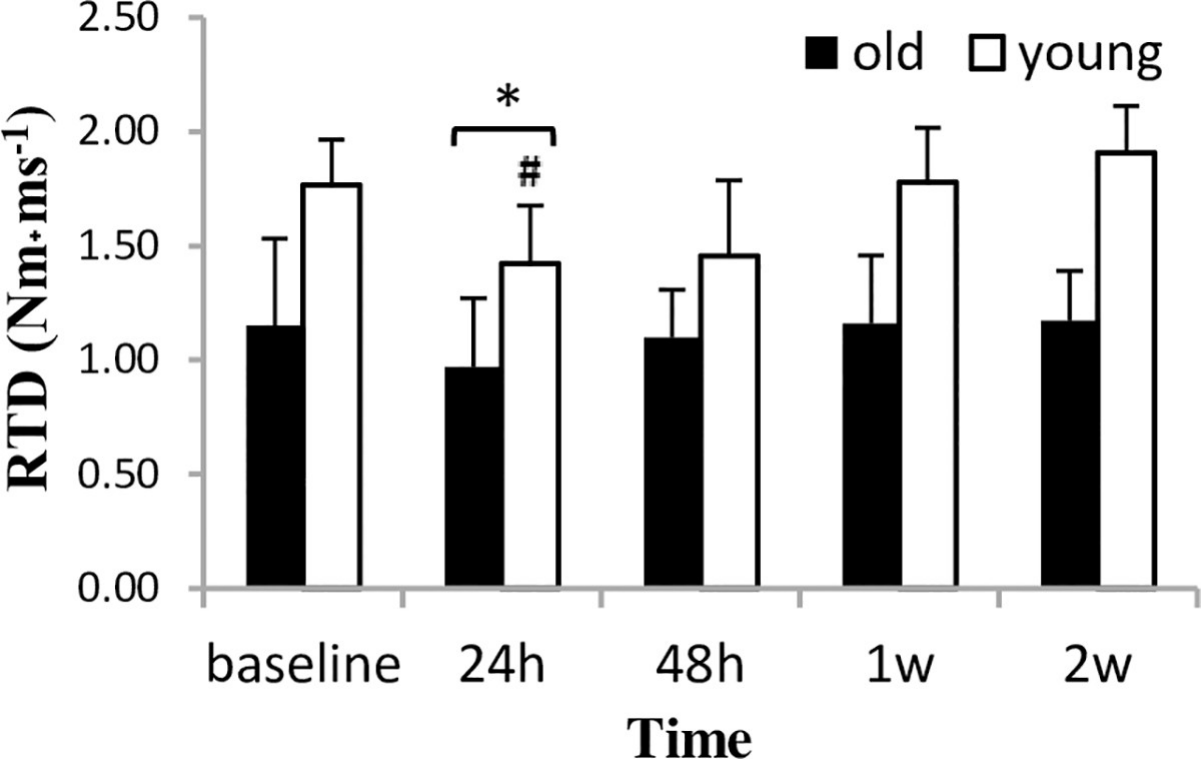
RTD of maximal voluntary isometric contraction at 70° of knee flexion (mean±SD) during a two-week eccentric-concentric knee extensor training in old and young men. RTD = rate of torque development. * Significantly different from baseline (p<0.05) after significant time-main effect. # Significant difference between groups (p<0.05).

A significant time main effect (F_4.15_ = 13.33, p = 0.000) and group by time interaction (F_4.15_ = 3.60, p = 0.030) was found for RTD (Fig 2). RTD was reduced only in the young group 24 h after bout 1 (p = 0.03), and tended to increase at the last test session (p = 0.090). Despite the significant time main effect, no change in RTD was observed for the old group during the experiment.

**Fig 3:**
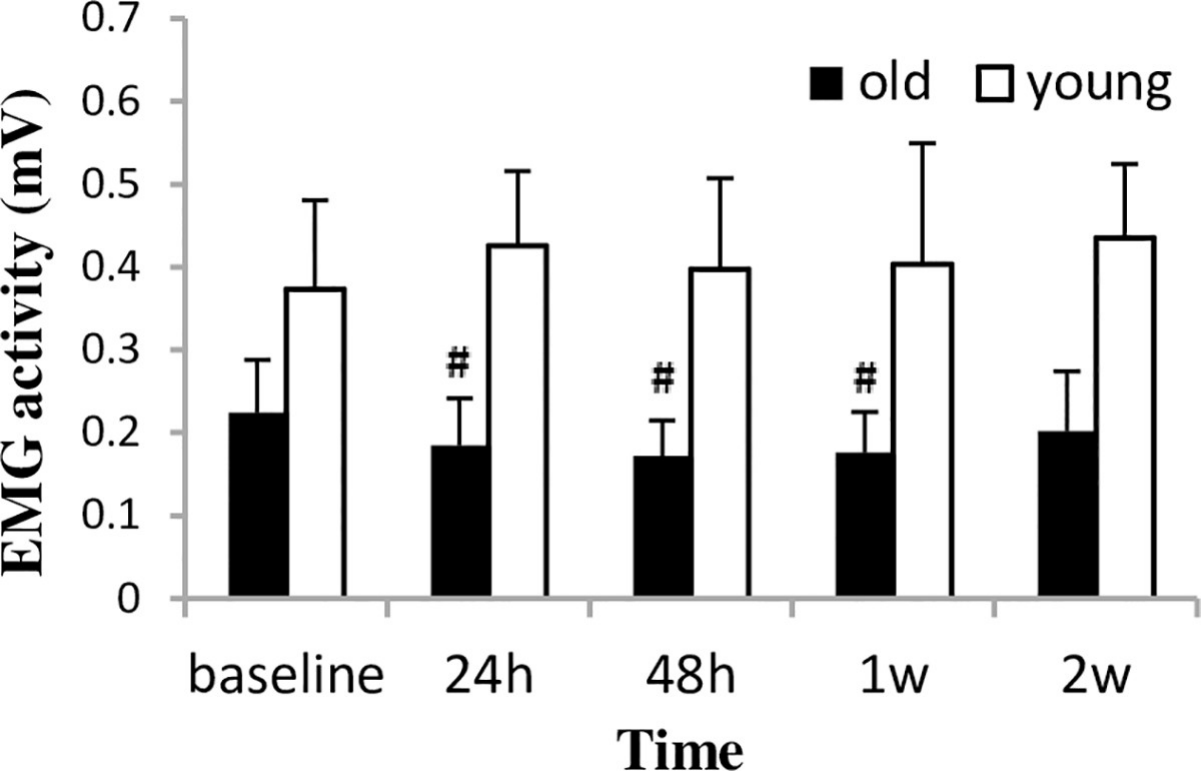
EMG activity (mean±SD) during a two-week eccentric-concentric knee extensor training in old and young men. EMG = electromyography. **#** Significantly different from baseline (p<0.05).

There was no significant time effect in EMG activity; however, the significant group by time interaction (F_4.15_ = 2.58, p = 0.047) suggests that the two groups responded differently (Fig 3). The post-hoc analyses revealed that EMG activity in the old group decreased at 24 h (p = 0.021) and only recovered by the end of the experiment. In contrast, EMG activity in the young group tended to increase from baseline to 24 h after bout 1 (p = 0.089); however, this elevation remained non-significant also at later measurement times.

**Fig 4:**
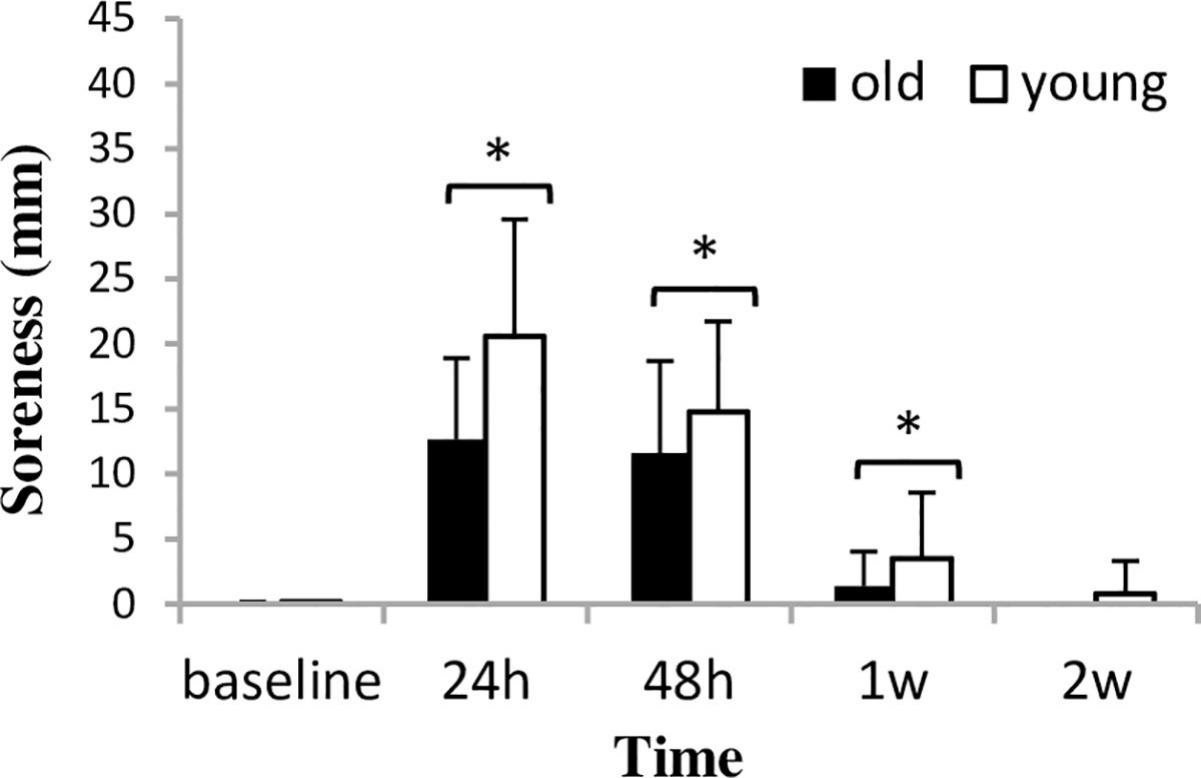
Muscle soreness (mean±SD) during a two-week eccentric-concentric knee extensor training in old and young men. * Significantly different from baseline (p<0.05) after significant time-main effect.

Muscle soreness peaked at 24 h after the first bout in both groups (significantly different from baseline, p = 0.000), and with further bouts, it gradually decreased and returned to the baseline level at the last test session (Fig 4). We found no between-group difference in muscle soreness at any of the measurement times.

**Fig 5:**
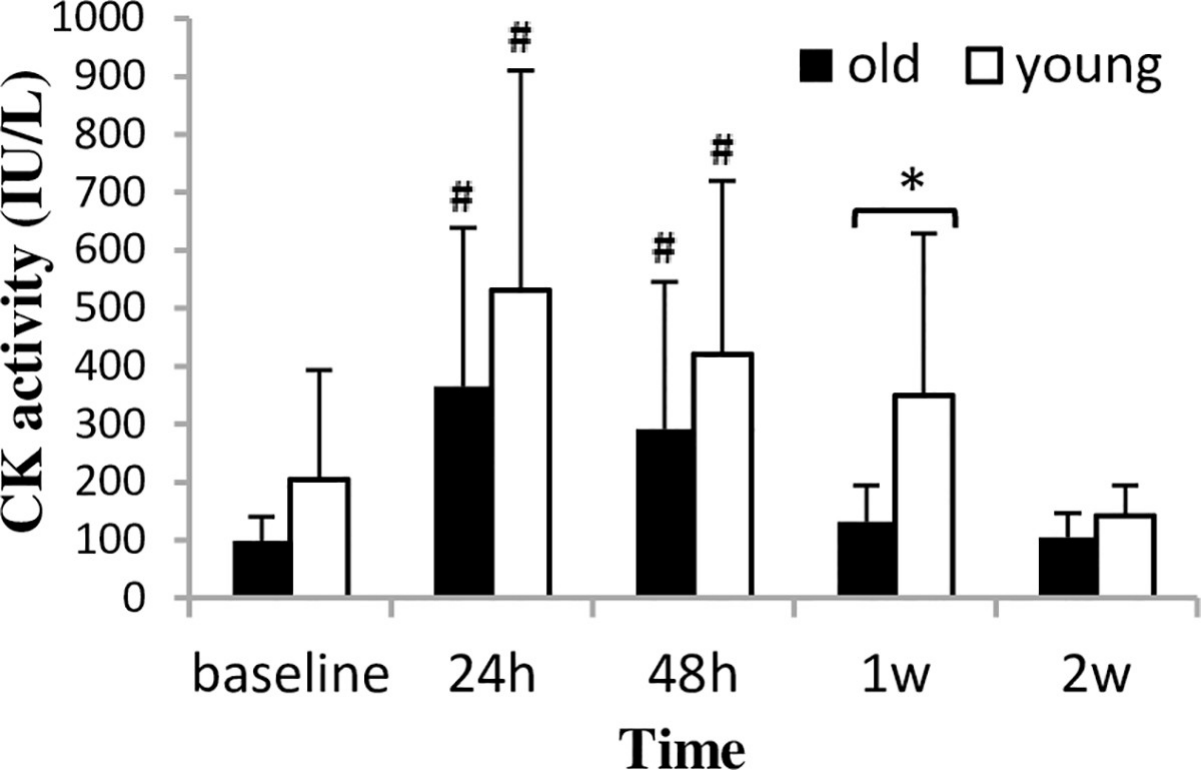
CK activity (mean±SD) during a two-week eccentric-concentric knee extensor training in old and young men. CK = creatine kinase. * Significant difference between groups (p<0.05) **#** Significantly different from baseline (p<0.05).

**Fig 6:**
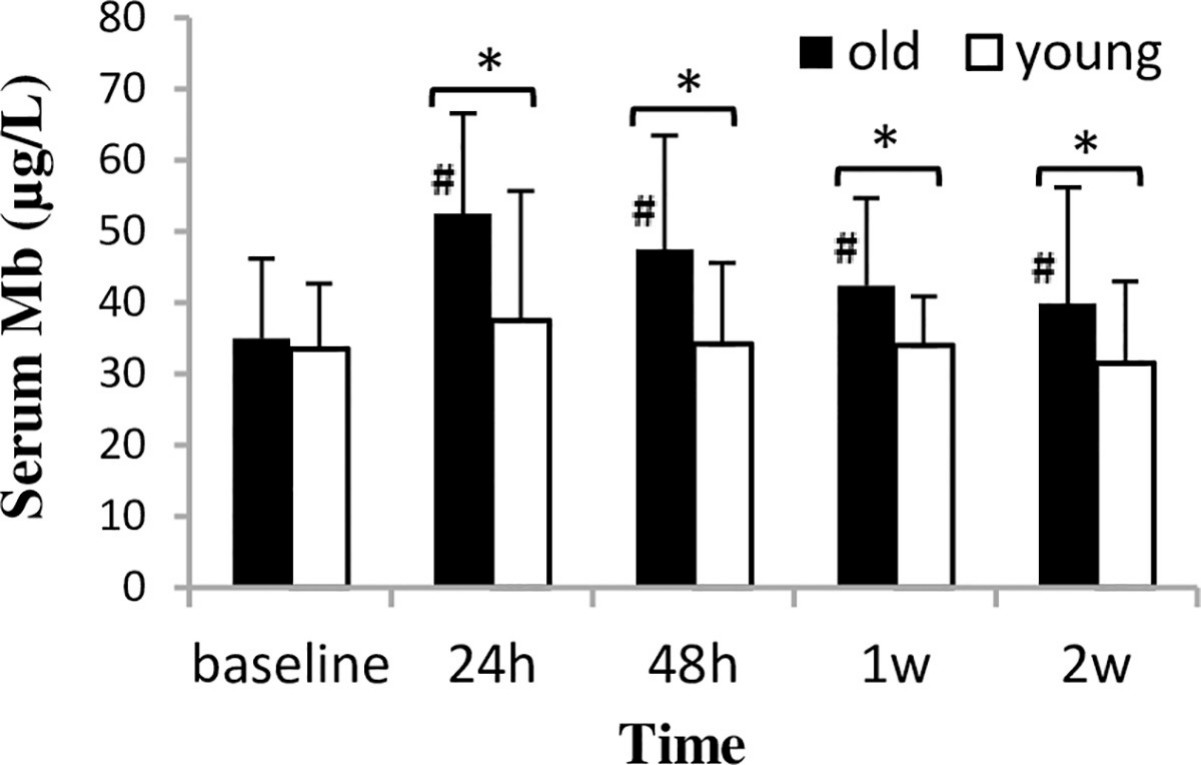
Mb concentration (mean±SD) during a two-week eccentric-concentric knee extensor training in old and young men. Mb = myoglobin * Significant difference between groups (p<0.05) # Significantly different from baseline (p<0.05).

CK activity peaked at 24 h after bout 1 (p = 0.001) and then gradually returned to the baseline level by the last test session in both groups (Fig 5). Between-group difference in CK activity was found only at 1 week after bout 1. Mb level was unchanged in the young group throughout the experiment (Fig 6). However, in the old group, Mb level increased significantly and peaked at 24 h after bout 1 (p = 0.001), and in the last test session it was still elevated (p = 0.039).

Table 2 shows the within-group percent changes in MVC torque, RTD, muscle soreness, CK, and Mb from baseline to 24 h, 48 h, week 1, and week 2. Between-group comparisons (using old men’s values expressed as percent of young) are also shown for 24h, 48h, week 1 and week 2 test periods.

**Table 2:** Percent change from baseline to 24h, 48h, 1w and 2w within each group, and between group comparisons (using old men’s values expressed as percent of young) at 24h, 48h, 1w and 2w.

		24h		48h		1w		2w	
		old	young	old	young	old	young	old	young
MVC torque	within	-15	-14	-12	-14	-10	-11	-3	-3
	between	77		80		73		73	
RTD	within	-16	-20	-4	-18	1	1	2	8
	between	54		68		49		38	
SOR	within	632	970	480	735	71	135	0	40
	between	57		78		75		60	
CK	within	265	161	191	106	32	72	1	-31
	between	54		56		140		66	
Mb	within	51	11	36	2	21	2	14	-6
	between	138		137		123		124	

MVC = maximal isometric voluntary torque

RTD = rate of torque development

SOR = muscle soreness level sensed during the MVC test contractions

CK = creatine kinase activity

Mb = myoglobin concentration

Table 3 shows the two-week changes in SSC function. The only significant group by time interaction was found for the Wssc^+^/Wcon ratio (F_1.18_ = 3.20, p = 0.041), and the post-hoc test revealed that by the last test session it increased significantly in the young group (p = 0.026) and remained unchanged in the old group. A significant time main effect was found for Tssc^+^ (F_1.18_ = 0.73, p = 0.039), which reduced uniformly in the two groups by the last test session. All other SSC properties were unchanged.

**Table 3:** Effects of two-week eccentric—concentric quadriceps exercise training on SSC function.

*Variables*	Old				Young				2-week % change and time main effect		Interaction
	Baseline		2 weeks		Baseline		2 weeks				
W_ssc+_ (J)	49.26	±12.62	52.34	±11.75	74.01	±14.69	80.75	±18.6	8%	p = 0.033	p = 0.402
W_ssc-_ (J)	85.24	±17.17	81.31	±14.71	109.89	±16.78	107.32	±17.17	-4.3%	p = 0.130	p = 0.745
W_ssc+_/W_con_	1.12	±0.32	1.02	±0.11	1.11	±0.19	1.19	±0.21	-0.9%	p = 0.850	p = 0.041
T_ssc-_ (ms)	231	±39	234	±64	234	±32	237	±32	1.2%	p = 0.758	p = 0.990
T_ssc+_ (ms)	290	±134	258	±107	207	±46	198	±60	-8.2%	p = 0.039	p = 0.644
T_ssc_ (ms)	521	±141	492	±156	441	±60	435	±85	-3.7%	p = 0.548	p = 0.684

Mean values (±SD) for the individual groups and percent change from baseline to two weeks for the two groups combined (time main effect) are presented.

SSC = stretch-shortening cycle

W_ssc+_ = mechanical work in the positive (concentric) phase of the SSC test contraction

W_ssc-_ = mechanical work in the negative (eccentric) phase of the SSC test contraction

W_ssc+_/W_con_ = ratio of the positive work in the SSC test contraction and the work in the pure concentric test contraction

T_ssc-_ = time to complete the negative (eccentric) phase in the SSC test contraction

T_ssc+_ = time to complete the positive (concentric) phase in the SSC test contraction

T_ssc_ = time to complete the entire SSC test contraction

## Discussion

Though the time course of change and isometric MVC torque shows a similar pattern, elastic energy storage and re-use improved selectively in the young group at the end of the two-week-long exercise intervention. Furthermore, we demonstrated that old men respond with lowered EMG activity, elevated Mb level, and without RTD deficit in the early phase (24 h after bout 1) of the exercise, compared with the young.

At baseline, the young men demonstrated 40% greater EMG activity, isometric torque, rate of torque development, and SSC function, while the Wssc^+^/Wcon ratio was similar in the young and old subjects. Age-associated losses in strength, not caused by neurological deficits or muscle disease, were seen as a change in isometric torque and EMG in the old group as reported by others [35,36,37]. The Wssc^+^/Wcon ratio represents the ability to store and re-use elastic energy during SSC and it is agreed that this property is less affected by ageing [38].

A single bout of eccentric-biased exercise induces myofibrillar ruptures, elevations of muscle proteins in serum, reduced voluntary force, and delayed onset muscle soreness on the subsequent day [18,39,40,41,42,43]. At 24 h after bout 1, our subjects demonstrated 15% decline in isometric MVC torque, muscle soreness developed, and CK activity was three-fold higher than at baseline and was consistent with previously reported data for young and old humans in similar protocols in which antigravitational muscles were exercised [7,11]. The impaired contractility is associated with failures in excitation-contraction coupling, eventually leading to loss of voluntary force. CK enzyme efflux from muscle tissue to serum is a normal response as the membrane ruptures or its permeability changes after vigorous exercise [44]. The aforementioned EIMD markers in our studies did not differentiate the acute responsiveness of young and old subjects, which is in agreement with the results of previous studies using the same markers [7]. However, the group by time interaction in EMG activity, Mb, and RTD suggests that the two groups responded differently to the first exercise bout. EMG activity reduced by 18% in the old group, while a non-significant trend to increase was seen in the young group 24 hours after bout 1. The acute gain in EMG activity is often explained with the compensation mechanism: when some fibres are irresponsive, increased motor unit synchronicity could be a compensation strategy to maintain force in the damaged muscle [45]. Muscle soreness is often suggested to inhibit central drive; however, this was not the case in our study because there was no difference in soreness between groups [46]. Perhaps the increase in EMG activity in the young group is due to a lack of full motor unit pool recruitment that improves following exercise (repeated test effect). Furthermore, we assume that in the old people the sarcolemma damage was more. Damage to the sarcolemma reduces the efficiency of the electrical conductivity and the strength of the contraction [47].

Twenty-four hours after bout 1, RTD was reduced only in the young group. RTD, which represents contractile speed, at least in part, relies on the activation of fast motor units. Because both fast motor unit content and their recruitment is reduced in the ageing muscle [48,49], we suggest that in the young subjects, the exercises used in our experiment probably damaged more fast motor units than in the old subjects. Furthermore, the power reduction was more than two fold lower in the older adults immediately after a bout of eccentric contractions, compared to young subjects [50], maybe due to the selective fatigue of fast motor units. Therefore, some fast motor units most likely became overloaded in our old subjects during the exercise because of fatigue or because they were not recruited at all preventing significant damage to the contractile units.

It is interesting in the present study that although CK responses were similar in the two experimental groups, Mb levels were elevated in the old group 24 hours after bout one. This result would occur if an aging-induced shift in muscle fiber type was present. Old humans demonstrate larger ratio of type I fibers [49], compared with young. Type I fibers contain more myoglobin than type II, however, type II fibers are more susceptible to injury. As a result, during microdamage, more myoglobin is released in the old people, however, CK activity and soreness tend to be greater in the young. On the other hand, Mb level is often considered a marker of sarcolemma vulnerability [49], therefore more sarcolemma can be ruptured after eccentric exercise in old people.

Finally, a potential factor for Mb release might be the transient decrease of energy (ATP) of the skeletal muscle cells during strenuous physical exercise. During muscle contractions, the initially low ATP turnover rate might increase 100-fold to maintain cellular ATP at constant level [51]. However, in the old group, the energy status of the muscle cells might not recover within 48 h, which shows the weaker metabolic adaptation capacity of the old subjects.

From 48 h after bout 1, the most-often-measured markers, isometric torque, CK, and soreness recovered uniformly in the two groups, which is in agreement with previous data found in young subjects showing weaker symptoms when exercise is continued [52]. A parallel change in isometric torque and EMG was observed in the old group, suggesting that, after the initial reduction, recovery of maximal force was mediated by gradually increasing neural drive. In contrast, the isometric torque recovery was accompanied with unchanged EMG activity in the young group, but it is important to note that EMG tended to be greater in test sessions 2 to 5 than the baseline. Therefore, in the early phase of a high force resistance exercise, young men either maintain normal neural drive or tend to show early favorable adaptation. In contrast, this mechanism is delayed in the old subjects, and adaptation may be expected beyond two weeks. Also, the slower EMG recovery in the old men can be explained by higher sarcolemmal damage, supported by the marked Mb elevation in the old subjects.

Despite the initial deficit, the young subjects recovered quickly in RTD and tended to improve by the end of the two-week protocol. In contrast, old subjects demonstrated no change in RTD during the entire period. Similarly, no adaptation in RTD was observed for the old subjects after 10 weeks of slow velocity eccentric exercise showing no adaptation in rapid contractility [4]. This lack of responsiveness in aging men can again be explained by the low number of fast motor units and/or their delayed recruitment with additional exercise bouts.

A novel observation in the present study is that after bout 1, Mb level remained elevated in the old group, despite the return of CK to normal levels. In contrast, Mb level was unchanged in the young group. In our opinion, persistent elevated Mb concentration might be an indicator of the extent of adaptability of skeletal muscle to physical exercise.

The data discussed above should be interpreted with caution. Studies of the repeated bout effect usually measured effects at 24 hours after bout 1 and bout 2 with the recovery of a few days or weeks between exercise bouts [18,40,52,53]. Instead of demonstrating the repeated bout effect, our aim was to measure the damage markers and EMG activity on days when an actual bout was to be performed (72 h after bout 3 and bout 6). Therefore, it is difficult to compare our results with those obtained from previous repeated bout effect studies because of the different measurement times. Instead, we evaluated the pre-bout physiologic status of our subjects rather than the acute post-bout responses during a realistic multi-bout exercise program.

Maintaining SSC function is important in the older adults because it is associated with muscle mechanical efficiency, movement economy, and fatigability [54]. In our study, the two-week adaptation to eccentric concentric exercise in SSC function was age-dependent and was specific to the SSC property measured.

The significant time main effect in positive work and the lack of significance in negative work suggest that concentric contractility improved selectively in both old and young subjects. Furthermore, the decrease in time to complete the concentric phase of the SSC would result in an increase in positive work observed.

Not surprisingly, the gain in positive work resulted from using maximal effort concentric muscle actions and, therefore, was not due to task specific effects. In addition, the concentric action in the exercise started immediately after the eccentric action, when the highest tension usually develops. Therefore, subjects initiated the concentric phase with maximally activated quadriceps, enhancing quick adaptation in concentric work. However, negative work was unchanged in both groups. Since our exercise protocols contain both type of contractions, the lack of improvement in an eccentric contractility was surprising. Generally, strength adaptation to eccentric exercise is more pronounced than strength gains due to concentric exercise as reported for long-term interventions [55,56,57]. It is possible that pain, which is more pronounced during eccentric action, prevented subjects from fully activating the quadriceps during the eccentric phase, resulting in insufficient stimulus for adaptation in eccentric work. Despite the fact that concentric contractility improved regardless of age, enhanced elastic energy storage and re-use (indicated by increased Wssc^+^/Wcon ratio) was shown only in young men at the end of the two-week protocol. The group by time interaction in the Wssc^+^/Wcon ratio can be explained by the fact that the older adults demonstrated high variability in this muscle property, which prevented the detection of significant improvements. Still, the adaptation in some of our old subjects was notable.

It is important to note that SSC function improved along with no change in isometric torque during the two-week experiment. It seems that the transfer effect of the present exercise was more favourable to SSC function versus isometric contractility, probably because of its dynamic nature. Adaptation in isometric versus dynamic contractility showed small sensitivity (6% vs. 23% change) in old humans even after 10 weeks of dynamic exercise [4], supporting our finding. In addition, short-term isometric torque adaptation in young humans was seen only after 3 days tapering from a vigorous exercise program (7 quadriceps exercise bouts within 8 days). Finally, elastic energy storage and re-use relies (in part) on the series elastic muscle components, which remain intact in the presence of myofibrillar damage [28], allowing faster adaptation in this mechanical property.

An important limitation in the present protocol was that SSC properties were measured only at test 1 (baseline) and test 5 (two weeks after bout 1); therefore, we have no information on how SSC function declines acutely and recovers in old and young subjects. However, in other studies, subjects have adapted quickly to SSC tests even when no additional exercise was performed [22]. Therefore, we omitted the SSC tests from test 2, 3, and 4.

Another limitation was that functional tests were not used to measure subjects’ mobility and functionality. However, the EIMD symptoms were small and the low sensitivity of functional tests would have probably prevented us from detecting changes in two weeks. Finally, delayed responses in common central and peripheral fatigue markers (voluntary activation, muscle contractility and membrane excitability, cortico-spinal excitability, and reflex response) using twitch interpolation and transcranial magnetic stimulation techniques should also be measured in order to reveal neuro-mechanical mechanisms responsible for the ‘secondary strength deficit’ and its recovery. Also, we were unable to measure motor unit firing frequency, which may change with ageing.

In summary, a two-week long high-intensity quadriceps exercise induced a similar time course of changes in isometric torque, CK activity and muscle soreness in young and old men. In contrast, the recovery was mediated by different myoelectric changes. In contrast with the young, RTD remained unchanged in the old subjects, probably because of the smaller amount of larger motor units and/or their de-recruitment. Finally, persistent elevations in Mb levels suggest higher sarcolemma vulnerability and less effective metabolic adaptation in the older adults, which, however, did not affect muscle contractility. Therefore, the design of exercise interventions to avoid overuse and loss of motivation and interest in older adults should be considered by gerontologists and strength specialists.

## Supporting information

S1 Video The stretch-shortening cycle (SSC) test using Multicont II dynamometer. (AVI)
